# Next generation sequencing and comparative analyses of *Xenopus* mitogenomes

**DOI:** 10.1186/1471-2164-13-496

**Published:** 2012-09-19

**Authors:** Rhiannon E Lloyd, Peter G Foster, Matthew Guille, D Timothy J Littlewood

**Affiliations:** 1 Institute of Biomedical and Biomolecular Sciences, University of Portsmouth, Portsmouth, PO1 2DT, UK; 2 Department of Zoology, Natural History Museum, Cromwell Road, London, SW7 5BD, UK; 3 Institute of Zoology, Zoological Society of London, Regent’s Park, London, NW1 4RY, UK

**Keywords:** Xenopus, Mitochondrial DNA, Next generation sequencing, Phylogeny, Mitogenomics, Comparative analyses, Variation, Selection and molecular markers

## Abstract

**Background:**

Mitochondrial genomes comprise a small but critical component of the total DNA in eukaryotic organisms. They encode several key proteins for the cell’s major energy producing apparatus, the mitochondrial respiratory chain. Additonally, their nucleotide and amino acid sequences are of great utility as markers for systematics, molecular ecology and forensics. Their characterization through nucleotide sequencing is a fundamental starting point in mitogenomics. Methods to amplify complete mitochondrial genomes rapidly and efficiently from microgram quantities of tissue of single individuals are, however, not always available. Here we validate two approaches, which combine long-PCR with Roche 454 pyrosequencing technology, to obtain two complete mitochondrial genomes from individual amphibian species.

**Results:**

We obtained two new xenopus frogs (*Xenopus borealis* and *X. victorianus*) complete mitochondrial genome sequences by means of long-PCR followed by 454 of individual genomes (approach 1) or of multiple pooled genomes (approach 2), the mean depth of coverage per nucleotide was 9823 and 186, respectively. We also characterised and compared the new mitogenomes against their sister taxa; *X. laevis* and *Silurana tropicalis*, two of the most intensely studied amphibians. Our results demonstrate how our approaches can be used to obtain complete amphibian mitogenomes with depths of coverage that far surpass traditional primer-walking strategies, at either the same cost or less. Our results also demonstrate: that the size, gene content and order are the same among xenopus mitogenomes and that *S. tropicalis* form a separate clade to the other xenopus, among which *X. laevis* and *X. victorianus* were most closely related. Nucleotide and amino acid diversity was found to vary across the xenopus mitogenomes, with the greatest diversity observed in the Complex 1 gene *nad4l* and the least diversity observed in Complex 4 genes (*cox1-3*). All protein-coding genes were shown to be under strong negative (purifying selection), with genes under the strongest pressure (Complex 4) also being the most highly expressed, highlighting their potentially crucial functions in the mitochondrial respiratory chain.

**Conclusions:**

Next generation sequencing of long-PCR amplicons using single taxon or multi-taxon approaches enabled two new species of *Xenopus* mtDNA to be fully characterized. We anticipate our complete mitochondrial genome amplification methods to be applicable to other amphibians, helpful for identifying the most appropriate markers for differentiating species, populations and resolving phylogenies, a pressing need since amphibians are undergoing drastic global decline. Our mtDNAs also provide templates for conserved primer design and the assembly of RNA and DNA reads following high throughput “omic” techniques such as RNA- and ChIP-seq. These could help us better understand how processes such mitochondrial replication and gene expression influence xenopus growth and development, as well as how they evolved and are regulated.

## Background

Metazoan cells are formed from a combination of nuclear (chromosomal) DNA and mitochondrial (extra-chromosomal) DNA (mtDNA). Animal mitochondrial genomes commonly include two ribosomal rRNAs, 22 tRNAs and 13 protein-coding genes. The latter gene-class encode for the proteins of the respiratory chain (RC), a multi-complex system (I to V), which in aerobic cells, transports electrons from NADH or FADH_2_ to molecular oxygen. This results in a proton gradient across the inner mitochondrial membrane that drives the synthesis of cellular energy (ATP). Mutations in mitochondrial genes and some of the 80 or so nuclear genes that make up the RC are associated with a broad range of diseases, ageing and cancer [1].

Mitochondrial genomes are of intrinsic importance for cellular function, but through their nucleotide and amino acid sequences are also of great utility as a source of markers for systematics and molecular ecology (e.g., [2]), and also in forensics (e.g., [3,4]). As their characterization is becoming easier and cheaper, increasing interest in comparative mitogenomics and the use of entire mtDNAs in systematics is gathering pace for some animal groups, particularly vertebrates; e.g. birds [5], mammals [6], fish [7] and amphibians. Currently, there are over 94 complete mtDNAs characterized for Amphibia (e.g. [8-11]), and many more for the other groups.

Amphibians colonized land ~350 million years ago and have since evolved into a wide variety of ecological and morphological types. Over 6,300 species of amphibians have been described to date, with the number of new species being discovered increasing annually [12]. Paradoxically, amphibian populations are undergoing a drastic global decline due to anthropogenic influences such as habitat destruction and pollution but also due to diseases such as the fungus *Batrachochytrium dendrobatidis* (e.g. see [13])*.* Thus, there is a pressing need to catalogue and monitor an ever-changing amphibian biodiversity, and to record fluctuations in species ranges as they are influenced by disease, environmental and ecological change. Many amphibian species can be morphologically similar over the course of their life cycles but molecular tools can aid in their identification, regardless of developmental stage. Typically, a relatively small sequence of mtDNA (a ‘DNA barcode’) encompassing part of one gene (e.g. *rrnL*; [14]) or a few genes (e.g. *cytb*, *rrnS* and *rrnL*; [15]) is used for resolving the identity and/or the phylogeny of amphibian species. DNA barcode efficacy depends upon a marker being able to differentiate between inter- and intra-specific variation and they are not always completely reliable [16]. Nucleotide diversity across the mitochondrial genomes of Metazoa is also highly variable [17], suggesting that molecular-based studies might benefit from the study of complete mtDNAs. Depending on goals and methodology, complete mtDNAs provide access to regions of high variation (useful for differentiating taxa, population genetics identifying individuals or species specific primer design), low variation (useful for universal primer design, alignment and resolving deeper phylogenies), or simply an opportunity to select from among all available sites to devise markers for a particular analysis or purpose (e.g. [18]).

Traditional approaches for sequencing them have required grams of tissue to extract and enrich sufficient quantities of ‘pure’ mtDNA (e.g. [19]). For small-bodied amphibians, this might necessitate the pooling of tissues from several individuals, increasing the chance of heterogeneous mtDNA variants. Also, trace amounts of nuclear DNA (nDNA) may remain in the ‘pure’ mtDNA, thus introducing the possibility of amplifying mitochondrial pseudogenes and introducing errors into the final sequence (see [20]). Enrichment for mitochondrial DNA can minimize the likelihood of amplifying mitochondrial pseudogenes [21], and targeted long-PCR based approaches are likely to avoid single, or short concatenated lengths of pseudogenes. Even if pure mtDNA is obtained, unless a suitable optimized primer set for the target species is available, primer-walking (the most common method used to obtain complete mitochondrial genomes to date) is time-consuming.

In this study we validate two similar approaches for rapidly and efficiently obtaining complete mitochondrial genomes from individual amphibian species. Starting with as little as one egg, both approaches combine long-PCR with next generation sequencing (Roche 454 pyrosequencing technology). Amplifying complete mtDNAs in a few overlapping fragments using long-PCR reduces the amount of starting material; we achieved complete coverage of mtDNAs with just two primer pairs. Our first approach is more costly and involves long-PCR followed by 454 sequencing of individual mtDNAs and generates high quality sequence data, with a very high depth of coverage per nucleotide (up to ~6000×; [22]). This amount of coverage is unnecessarily high for most applications, thus a second approach that involves long-PCR followed by 454 of multiple pooled mtDNAs was also validated [23], offering a better balance between cost and data quality. The depth of coverage per nucleotide obtained using the latter approach still far exceeds that commonly obtained by primer-walking (typically by > 15×; [23]).

We chose to validate the two approaches for amphibians in general using material from *Xenopus*, in part due to ease of access to material but also due to their popularity as a model organism for understanding vertebrate growth and development (reviewed in [24]). Here we use the term ‘xenopus’ as a common noun for frogs in the genera *Xenopus* and *Silurana*, sister taxa that were until recently combined in a single genus. Two new complete *Xenopus* mitochondrial genomes were obtained; *Xenopus borealis* and *X. victorianus.* The latter represents the first mitochondrial genome obtained from the next generation sequencing of so many (>450) pooled long-PCR amplicons. Two existing xenopus sequences (*X. laevis* and *Silurana tropicalis*) were used to design the ‘universal’ primers for the long-PCR and to annotate the features of the new genomes, and used as a basis for comparative analyses. Although xenopus are the most intensively studied amphibians, providing insights into cellular reprogramming, organogenesis, regeneration, gene regulatory networks and protein interactions [24], the role of mitochondrial DNA (mtDNA) in these processes has received relatively little attention. Characterizing mtDNAs of individual species and conducting comparative mitogenomic analyses are important first steps in developing this knowledge further. We analysed xenopus mtDNAs in terms of simple descriptors and pairwise comparisons involving measures of variation and selection to explore further the utility of mitochondrial genomes in xenopus research. Species of *Xenopus* are also all notably polyploid, which renders nuclear gene markers less suitable for reconstructing phylogenies. In this context we assessed the suitability of complete mitochondrial genomes alone in resolving xenopus phylogeny, as well as the suitability of existing mtDNA barcodes for differentiating xenopus species, populations and individuals.

## Results and discussion

### Verification of long-PCR amplicon identity and primer region sequences

**Figure 1:**
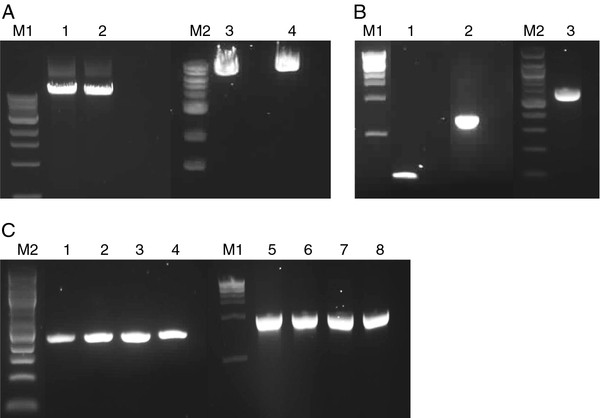
**Long PCR, COX1, 16S, primer region 1 and primer region 2 amplicons.** Agarose gel electrophoresis of (**A**) *Xenopus borealis* (XB; lanes 1 and 2) and *X. victorianus* (XV; lanes 3 and 4) PCR fragments using Long F1/R2 (lanes 1 and 3) and Long F2/R1 primers (lanes 2 and 4). (**B**) XB (lanes 1 and 2) and XV (lane 3) PCR fragments using COX1 (lane 1) and 16SA-Lmod/H (lanes 2 and 3) primers. (**C**) XB (lanes 1-2 and 5-6) and XV (lanes 3-4 and 7-8) PCR fragments using AMP1F/R (lanes 1-4) and AMP2F/R (lanes 5-8) primers. M1 and M2 = 1kb and 100bp DNA ladders, respectively.

The complete mitochondrial genome of one female each of *X. borealis* (XB) and *X. victorianus* (XV) was obtained by long-PCR amplification of 10 ng of egg DNA in two adjacent amplicons. Amplicon 1 was ~8,000 bp and amplicon 2 was ~9,500 bp in size, as predicted from published mtDNAs of xenopus frogs (Figure 1). Typically, each long-PCR reaction yielded 5 μg of each amplicon, as determined via the Picogreen assay. Conventional PCR amplification of amplicon 1 with *rrnL* and/or *cox1* primers generated ~580bp and 190bp fragments, respectively (Figure 1). The sequence obtained from the *rrnL* fragment was 100% identical to that deposited in the NCBI nucleotide database for *Xenopus borealis*, confirming the specific identity of the XB sample used in this study. Sequences of *cox1* for XB are absent from the database. Nonetheless, the sequence obtained using the *cox1* primers was 80% and 81% identical to corresponding regions found within the *X. laevis* (XL) and *Silurana tropicalis* (ST) mitochondrial genomes. Reference sequences for *rrnL* of XV were also absent from the database, but the sequence obtained shared a 98% nucleotide identity with the corresponding region in the XL mitochondrial genome. Since the two long-PCR amplicons were adjacent, rather than overlapping, fragments (294bp and 912bp) containing the LongF1/R1 and Long F2/R2 primer regions, respectively, were amplified (Figure 1) and sequenced. Each primer region was 100% identical to the corresponding regions found within the appropriate *Xenopus* mitochondrial genome derived by 454 (this study).

### Automated sequencing and assembly of the *Xenopus* mitochondrial genomes

**Figure 2:**
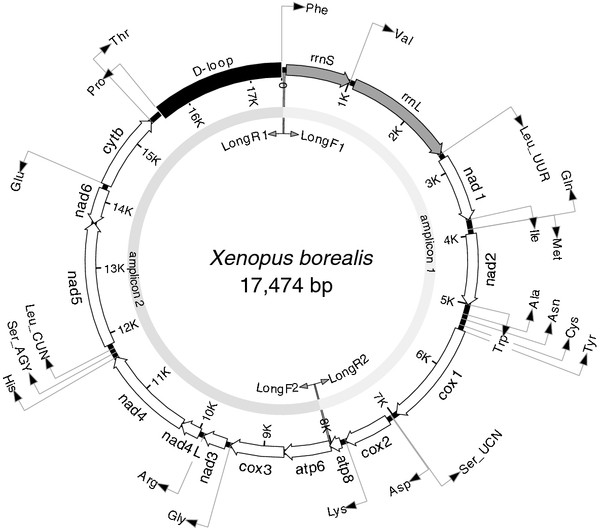
***Xenopus borealis* mitochondrial genome.** The complete mitochondrial genome of *Xenopus borealis* (17,474 bp, drawn to scale) All 13 protein coding genes are shown as open arrows, 2 ribosomal RNAs as shaded arrows and 22 tRNAs as arrowed lines. Each tRNA is shown by its single letter amino acid code. The two leucine and two serine tRNAs are differentiated by their respective anti-codons. The direction of transcription is indicated by the arrows. Also shown is the non-coding D-loop (control region, black) and the position of the primers (LongF1/R2 and LongF2/R1) used to generate the two long-PCR amplicons, which were pooled and sequenced using 454 technology.

The complete mitochondrial genome sequences of *Xenopus borealis* (XB; GenBank accession no. JX155859) and *X. victorianus* (XV; GenBank accession no. JX155858) were 17,474 and 17,716 bp in size, respectively (Tables 1 and 2, Figure 2), thus similar in size to the two published *Xenopus* sequences (XL and ST: 17,552 and 17,619 bp, respectively). The XB and XV mitochondrial genomes were assembled from 499,995 and 9,864 reads respectively; see Table 1. The mean read lengths were ~535 (XB) and ~862 (XV) nucleotides and the total data contributing to the assembled sequences was ~171.6 Mb (XB) and ~7.6 Mb (XV) (Table 1). Mean depth of coverage (DOC) for every nucleotide position ranged from 4923-32030 (XB) and 1-643 (XV) (Table 1), and the mean DOC over the entire mitochondrial genome was ~9823 (XB) and ~186 (XV).

**Table 1:** **Consensus sequence length and read statistics for the *Xenopus borealis *and *X. victorianus *mitochondrial genomes obtained using 454**

**Species**	**Total sequence length (nt)**	**Read output**	**Reads mapped (%)**	**Total sequence output (nt)**	**Total sequence mapped (%)**	**Read length (nt)**	**Mean read length (SD)**	**DOC/nt**
*X. borealis*	17474	499995	489725 (97.95%)	172077728	171646577 (99.75%)	57-1201	535.79 (111.3)	4923-32030
*X. victorianus*	17716	9864	6627 (67.18%)	8500481	3432828 (40.40%)	324-1401	861.77 (196.86)	1-643

Abbreviations: Nt: nucleotide. SD: standard deviation. %: percentage. DOC: depth of coverage.

**Table 2:** **Length and position of genes in the mitochondrial genomes of *Xenopus***

		***Xenopus laevis***								***Silurana (X.) tropicalis***								***X. borealis***								***X. victorianus***							
**Feature**	**Description**	**Position**		**Length**	**Start/Stop**	**A (%)**	**C (%)**	**G (%)**	**T (%)**	**Position**		**Length**	**Start/Stop**	**A (%)**	**C (%)**	**G (%)**	**T (%)**	**Position**		**Length**	**Start/Stop**	**A (%)**	**C (%)**	**G (%)**	**T (%)**	**Position**		**Length**	**Start/Stop**	**A (%)**	**C (%)**	**G (%)**	**T (%)**
		**(nt)**		**(nt)**						**(nt)**		**(nt)**						**(nt)**		**(nt)**						**(nt)**		**(nt)**					
tRNA	Phe	1	69	69		33.3	23.2	24.6	18.9	1	68	68		38.2	22.1	22.1	17.6	1	68	68		36.8	22.1	23.5	17.6	1	68	68		36.8	22.1	23.5	17.6
rRNA	rrnS	70	888	819		32.5	25.5	19.9	22.1	69	1011	943		32.7	27.8	20.0	19.5	69	1015	947		32.2	25.0	19.6	23.2	69	1016	948		32.3	25.9	19.6	22.2
tRNA	Val	889	957	69		34.8	24.6	13.0	27.6	1012	1081	70		32.9	28.6	14.3	24.2	1016	1085	70		32.9	24.3	14.3	28.5	1017	1085	69		33.3	23.2	14.5	29.0
rRNA	rrnL	958	2588	1631		36.4	21.0	17.7	24.9	1082	2716	1635		34.9	24.1	18.5	22.5	1086	2720	1635		36.5	19.6	18.2	25.7	1086	2720	1635		37.1	21.4	17.7	23.8
tRNA	Lee (UUR)	2589	2663	75		24.0	26.7	24.0	25.3	2717	2791	75		25.3	25.3	22.7	26.7	2721	2795	75		25.3	26.7	22.7	25.3	2722	2796	75		21.3	29.3	25.3	24.1
Gene	nad1	2664	3635	972	ATG/TAG	31.6	24.9	12.3	31.2	2792	3759	968	ATG/TAG	28.0	30.0	13.7	28.3	2796	3764	969	ATG/TAG	29.1	21.3	15.3	34.3	2801	3769	969	ATG/TAG	30.5	26.5	12.4	30.6
tRNA	Ile	3635	3705	71		31.0	21.1	22.5	25.4	3760	3830	71		28.2	23.9	25.4	22.5	3764	3834	71		31.0	21.1	22.5	25.4	3769	3839	71		31.0	22.5	22.5	24.0
tRNA	Gln [C]	3705	3775	71		21.1	14.1	31.0	33.8	3830	3900	71		21.1	14.1	31.0	33.8	3834	3904	71		21.1	12.7	31.0	35.2	3840	3909	70		20.0	14.3	13.4	52.3
tRNA	Met	3775	3843	69		31.9	26.1	14.5	27.5	3900	3968	69		31.9	24.6	14.5	29.0	3904	3972	69		33.3	23.2	14.5	29.0	3909	3977	69		31.9	26.1	14.5	27.5
Gene	nad2	3844	4881	1038	ATG/TAG	31.8	26.9	10.2	31.1	3969	5004	1036	ATG/TAG	29.5	33.3	9.7	27.5	3973	5010	1038	ATG/TAG	31.5	24.2	11.4	32.9	3978	5015	1038	ATG/TAG	32.2	27.5	9.3	31.0
tRNA	Trp	4880	4948	69		36.2	28.3	18.8	16.7	5005	5073	69		33.3	23.2	23.2	20.3	5009	5077	69		36.2	20.3	17.4	26.1	5014	5082	69		36.2	21.7	17.4	24.7
tRNA	Ala [C]	4951	5019	69		30.4	13.0	20.3	36.3	5077	5145	69		29.0	13.0	21.7	36.3	5084	5154	71		31.0	11.3	19.7	38.0	5084	5154	71		28.2	12.7	23.9	35.2
tRNA	Asn [C]	5021	5091	71		23.9	15.5	28.2	32.4	5147	5219	73		24.7	17.8	26.0	31.5	5154	5228	75		25.3	20.0	26.7	28.0	5154	5228	75		24.0	16.0	30.7	29.3
tRNA	Cys [C]	5190	5259	70		25.8	22.7	28.8	22.7	5258	5323	66		25.8	22.7	31.8	19.7	5255	5320	66		24.2	25.8	28.8	21.2	5260	5325	66		24.2	22.7	30.3	22.8
tRNA	Tyr [C]	6817	6887	71		21.4	20.0	31.4	27.2	5324	5393	70		20.0	22.9	31.4	25.7	5321	5392	72		23.6	26.4	27.8	22.2	5326	5397	72		22.2	22.2	29.2	26.4
Gene	cox1	5262	6816	1555	ATG/AAT	28.6	22.4	16.5	32.5	5395	6951	1557	GTG/TAA	27.3	25.1	17.5	30.1	5392	6948	1557	GTG/TAA	28.1	21.1	17.3	33.5	5397	6953	1557	GTG/TAA	28.5	23.3	16.1	32.1
tRNA	Ser (UCN) [C]	6817	6887	71		26.8	16.9	28.2	28.1	6954	7024	71		25.4	15.5	28.2	30.9	6950	7020	71		28.2	18.3	25.4	28.1	6956	7026	71		28.2	25.4	16.9	29.5
tRNA	Asp	6903	6971	69		31.9	21.7	23.2	23.2	7040	7108	69		37.3	26.1	18.8	17.8	7031	7099	69		37.7	15.9	14.5	31.9	7042	7109	68		38.9	22.1	19.1	19.9
Gene	cox2	6974	7661	688	ATG/T	32.1	24.4	14.7	28.8	7111	7798	688	ATG/T	31.4	28.2	15.1	25.3	7102	7789	688	ATG/T	32.3	21.9	14.8	31.0	7112	7799	688	ATG/T	32.3	25.0	14.2	28.5
tRNA	Lys	7662	7736	75		32.0	24.0	20.0	24.0	7799	7872	74		31.1	28.4	21.6	18.9	7790	7863	74		29.7	25.7	23.0	21.6	7800	7874	75		29.3	22.7	12.7	35.3
Gene	atp8	7738	7905	168	ATG/TAA	38.1	28.0	8.3	25.6	7874	8041	168	ATG/TAA	37.5	29.2	9.5	23.8	7865	8032	168	ATG/TAA	37.5	26.2	8.3	28.0	7876	8043	168	ATG/TAA	36.9	28.0	8.3	26.8
Gene	atp6	7896	8576	681	ATG/TAA	30.2	25.4	10.4	34.0	8032	8714	683	ATG/TAA	25.3	33.2	11.4	30.1	8023	8706	684	ATG/TAA	28.8	24.3	11.5	35.4	8034	8717	684	ATG/TAA	29.1	27.0	10.5	33.4
Gene	cox3	8576	9356	781	ATG/T	29.8	24.2	15.7	30.3	8715	9498	784	ATG/T	26.5	30.2	16.2	27.1	8706	9489	784	ATG/T	26.4	22.8	17.3	33.5	8717	9500	784	ATG/T	28.6	25.8	15.8	29.8
tRNA	Gly	9357	9426	70		27.1	18.6	14.3	40.0	9499	9568	70		38.6	21.4	14.3	25.7	9490	9559	70		35.7	21.4	14.3	28.6	9501	9570	70		35.7	18.6	15.7	30.0
Gene	nad3	9427	9769	343	ATG/TAA	26.2	26.2	13.7	33.9	9569	9911	343	ATG/AAT	24.8	30.6	14.3	30.3	9560	9902	343	ATG/T	24.5	22.4	16.9	36.2	9571	9913	343	ATG/T	25.1	28.6	12.8	33.5
tRNA	Arg	9770	9838	69		34.8	18.4	15.9	30.9	9912	9980	69		33.3	24.6	17.4	24.7	9903	9972	70		35.7	21.4	15.7	27.2	9914	9983	70		35.7	18.6	15.7	30.0
Gene	nad4L	9839	10135	297	ATG/TAA	30.3	23.9	12.8	33.0	9981	10277	297	ATG/TAA	23.9	33.0	15.5	27.6	9972	10268	297	ATG/TAA	26.6	24.6	13.8	35.0	9983	10279	297	ATG/TAA	26.6	27.3	13.1	33.0
Gene	nad4	10129	11512	1384	ATG/T	32.7	24.6	11.1	31.6	10271	11648	1378	ATG/T	29.0	31.5	11.5	28.0	10262	11639	1378	ATG/T	30.5	25.1	11.5	32.9	10273	11650	1378	ATG/T	31.8	26.1	11.1	31.0
tRNA	His	11513	11580	68		35.3	17.6	16.2	30.9	11649	11717	69		36.2	20.3	15.9	27.6	11640	11708	69		30.4	15.9	20.3	33.4	11651	11719	69		34.8	17.4	17.4	30.4
tRNA	Ser (AGY)	11581	11645	65		23.1	24.6	21.5	30.8	11718	11785	68		23.5	27.9	25.0	23.6	11708	11774	67		22.4	23.9	23.9	29.8	11719	11785	67		22.4	23.9	23.9	29.8
tRNA	Leu (CUN)	11646	11719	74		33.8	20.3	21.6	24.3	11786	11858	73		32.9	21.9	20.5	24.7	11774	11847	74		32.4	16.2	23.0	28.4	11785	11858	74		32.4	20.3	21.6	25.7
Gene	nad5	11720	13534	1815	ATG/TAA	33.2	23.3	11.5	32.0	11859	13676	1818	ATG/TAA	30.5	30.2	12.7	26.6	11848	13665	1818	ATG/TAA	31.4	23.7	12.6	32.3	11859	13676	1818	ATG/TAA	32.3	24.4	11.7	31.6
Gene	nad6 [C]	13530	14042	513	ATG/AGA	19.9	9.4	28.1	42.6	13668	14186	519	ATG/TAA	15.2	10.4	35.1	39.3	13657	14175	519	ATG/TAA	19.5	11.9	29.5	39.1	13668	14186	519	ATG/TAA	19.8	9.4	28.7	42.1
tRNA	Glu [C]	14043	14111	69		24.6	14.5	26.1	34.8	14187	14255	69		26.1	14.5	26.1	33.3	14176	14245	70		28.6	11.4	21.4	38.6	14187	14257	71		23.9	14.1	25.4	36.6
Gene	cytb	14114	15253	1140	ATG/TAG	29.5	25.4	12.6	32.5	14258	15400	1143	ATG/TAG	27.4	30.4	14.4	27.8	14246	15388	1143	ATG/TAG	27.7	23.3	14.8	34.2	14258	15400	1143	ATG/TAG	28.6	26.2	13.1	32.1
tRNA	Thr	15253	15322	70		30.0	22.9	20.0	27.1	15400	15470	71		26.8	26.8	22.5	23.9	15388	15458	71		26.8	25.4	21.1	26.7	15400	15471	72		29.2	25.0	20.8	25.0
tRNA	Pro [C]	15350	15418	69		21.7	11.6	38.4	28.3	15500	15566	67		22.4	11.9	29.9	35.8	15499	15565	67		20.9	11.9	31.3	35.9	15497	15563	67		23.9	10.4	28.4	37.3
Cont region	D-loop	15419	17552	2134		39.3	17.9	9.4	33.4	15567	17610	2044		39.0	20.2	10.7	30.1	15566	17474	1909		37.2	19.0	9.8	34.0	15564	17716	2153		39.3	19.0	9.1	32.6

Initiation and termination codons and base contents (%) are also indicated. Gene transcribed in the reverse (complementary) direction are indicated with [C].

### Annotation and characterisation of the *Xenopus* mitochondrial genomes

#### Length, gene content and order

Table 2 compares the full mitogenomes of the four xenopus species, indicating considerable conservation in gene content, size and arrangement. The small differences in size between the xenopus mitochondrial genomes (~250 bp) largely relates to an expansion of the D-loop, which is 1909 bp in XB, +225 bp in XL, +135 bp in ST and +244 bp in XV.

The gene content and order is the same for all four xenopus mitochondrial genomes in having 13 protein coding genes (the cytochrome c oxidase subunits 1–3 (*cox1-cox3*), the nicotinamide dehydrogenase subunits 1–6 (*nad1-nad6* and *nad4L*), cytochrome b (*cytb*) and adenosine triphosphatase subunits 6 and 8 (*atp6* and *atp8*), 22 transfer RNAs (tRNAs) and the small (*rrnS*) and large (*rrnL*) ribosomal RNAs (Table 2 and Figure 2). All protein coding genes, apart from *nad6*, are predicted to be transcribed from the same strand and in the same direction.

Since the gene order and content of mitochondrial genomes is thought to be reflective of phylogenetic relationships, with such features changing relatively rarely between closely related taxa [8], it is not surprising they are identical for the four xenopus mitochondrial genomes. The gene order and content of the four xenopus mitochondrial genomes is also typical of that found in vertebrates (e.g. human, bovine and mouse; [25]), as is often the case for “Archaeobatrachian” (primitive) anurans like xenopus [8,10,25] but not “Neobatrachian” (more derived) anurans [19].

In higher eukaryotes, the H-strand and L-strands each contain a distinct replication origin (OH and OL). H-strand replication begins in the D-loop, whereas L-strand replication does not begin until approximately two-thirds of the H-strand has been replicated. The ST, XB and XV D-loops each contained a sequence 75-90% similar to the XL OH sequence (nt 16980-17021). Similarly, the ST, XB and XV mitochondrial genomes each contained a sequence 79-91% similar to the OL sequence (nt 5092-5128). These regions likely represent the replication origins in these species.

#### Base-pair composition, codon usage and amino acid propensity

The mean GC content for all xenopus H-strand protein-coding genes was similar (39.3% ± 3.6), as was the asymmetric usage of the four base pairs between the H- and L-strands, i.e. the GC skew is -0.32 (G is preferentially located on the L-strand) and the AT skew is -0.02 (with more A in L-strand); see Table 3. Like other Chordata, xenopus H-strand protein coding genes are relatively GC-rich when compared to the following groups e.g. Annelida, Arthropoda, Cnidaria, Echinodermata, Mollusca, Platyhelminthes and Porifera that all have lower GC-means (range 23.64% to 38.18%). Surprisingly, Xenopus H-strand protein coding gene GC-richness in this study is most similar to that reported for Mammalia and Testudines (40.13 and 38.83, respectively) rather than that reported for Amphibia (37.45), previously. Calculating GC and AT skew indices [26] revealed that xenopus H-strand protein coding genes show an asymmetric distribution of the four bases between the H and L-strands, like many other metazoans [27]. Unusual among Metazoa, the GC and AT asymmetries observed in xenopus H-strand protein coding genes were both negative (a negative GC-skew and a positive AT-skew is more usual). However, the GC-skew (-0.32) observed in xenopus H-strand coding genes is more pronounced than the AT-skew (-0.02), typical among Metazoans. Such asymmetry is thought to be positively correlated with how long the H-strand remains single stranded during replication, increasing the time it is exposed to mutation [28]. On this basis, the mutation rate in xenopus H-strand protein coding genes could well be different to that observed in other amphibians (-0.25; [27]).

**Table 3:** GC and AT-skew indices

	**Mean A + T**	**AT**	**Mean C + G**	**GC**
	**(%)**	**skew**	**(%)**	**skew**
XL	62.4	−0.01	37.6	−0.32
ST	56.2	0.01	43.8	−0.37
XB	62.7	−0.06	37.3	−0.25
XV	61.3	−0.02	38.7	−0.35
ALL	60.7	−0.02	39.3	−0.32
SD	3.4	−0.3	3.6	−0.6

Percentage mean ± standard deviation (SD) base-pairs in xenopus H-strand protein-coding genes. Skew (or “asymmetry”) between base-pairs was calculated as follows: GC skew = (G – C)/total(GC) and AT skew = (A – T)/total(AT).

The codon usage was identical to that of other vertebrate mitogenomes, including other amphibians [29]: all 13 of the xenopus protein coding genes use ATG as an initiation codon, with the exception of the *cox1* gene in ST, XB and XV that uses either TAA or GTG. The most frequent termination codons used by xenopus mitogenomes were TAR and AGR, again characteristic of vertebrate/amphibian mitogenomes [29]. The next most frequently used termination codon was incomplete, a single nucleotide T, where the post-transcriptional polyadenylation is thought to complete a TAA termination codon, as suggested for amphibians and humans ( [29,30], respectively).

#### Phylogenetic analysis

**Figure 3:**
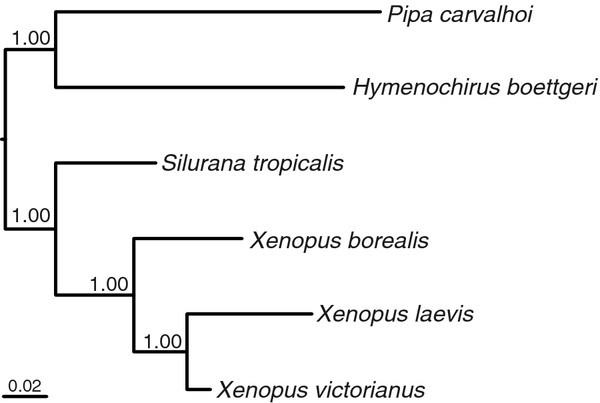
**Phylogenetic estimates of the interrelationship of four xenopus species and two relatives based on Bayesian analysis of amino acids from concatenated protein coding sequences.** Nodal support is given by posterior probabilities; branch-length scale indicates number of substitutions per site.

From the published mtDNAs available on GenBank, two taxa were selected as suitable outgroups: *Hymenochirus boettgeri* (NC_015615) and *Pipa carvalhoi* (NC_015617). Both are members of the Pipinae, sister to the Xenopodinae (the *Xenopus* and *Silurana* species) and their mtDNAs were published as part of a phylogenetic study of tongueless frogs by [31]. The full alignment of protein-coding genes consisted of 3,782 amino acids, with few indels (48 gaps in total, 6 taxa), and was deemed unambiguously aligned; of the aligned sites, 2,722 (73.3%) were identical. The resulting Bayesian phylogenetic analysis of concatenated protein coding genes, analysed as amino acids, is shown in Figure 3. Each node is supported unequivocally with maximal nodal support (100% posterior probabilities). Amongst the ingroup, the Xenopodinae, *Silurana* (ST) was resolved as sister to a monophyletic clade of *Xenopus*, within which *X. laevis* and *X. victorianus* were resolved as sister taxa. These results are consistent with other recently published phylogenies; e.g. one that used a 2335 bp region of mitochondrial DNA (including the *rrnS*, *trnV* and *rrnL* genes analysed as nucleotides; [32]) and one that used *cytb*, *rrnL* and *rrnS* and several nuclear genes as markers (e.g. recombination-activating gene 1 (RAG1)), also analysed as nucleotides [15]. The strong nodal support within the phylogeny suggests that many more species might be accommodated in an analysis of Xenopodinae (and Pipidae) using all mitochondrial protein coding genes. Currently, there are 15 species of *Xenopus* and 4 species of *Silurana* considered valid within this subfamily [32,33]. The genera are differentiated based on their chromosome complements. In *Silurana* chromosome numbers are multiples of 20 (1 diploid species and 3 tetraploid), in *Xenopus* chromosome numbers are multiples of 18, with all species polyploid (e.g. see [34]). Extant species are distributed across Africa but fossil forms from Brazil and Argentina suggest a possible Gondwanan origin of the Pipidae, and other fossils from Africa demonstrate a considerably wider historical distribution in Africa [32]. A mitogenomic approach to the systematics of Xenopodinae may provide additional insights into their evolutionary origins and patterns of radiation.

#### Sliding window analysis

**Figure 4:**
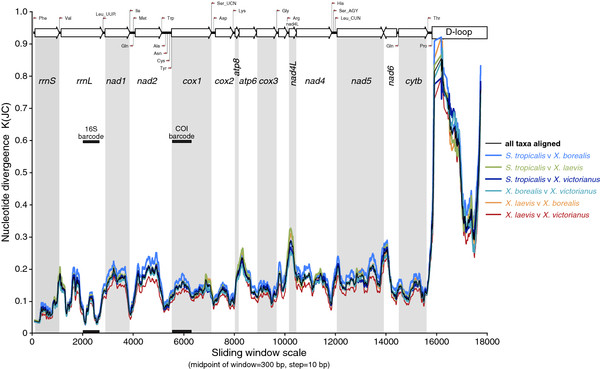
**Sliding window analysis of complete mitochondrial genome sequences of xenopus frogs.** The coloured lines show the value of nucleotide divergence K(JC) (average number of nucleotide substitutions per site between species with Jukes and Canor correction) in a sliding window analysis of window size 300 bp with step size 10 for: all four xenopus (black), ST v XL (green), ST v XB (light blue), ST v XV (dark blue), XL v XB (orange), XB v XV (turquoise) and XL and XV (red). Gene boundaries and primers and regions commonly used in DNA barcoding amphibians are indicated.

The four complete xenopus mitochondrial genomes were aligned in their entirety, at the nucleotide level, to estimate nucleotide divergence K(JC) (average number of nucleotide substitutions per site between species with Jukes Cantor correction) across the genome as revealed by sliding window analysis using DnaSP [35]; high values of K(JC) indicate greater sequence differences. Greatest divergence was observed in the comparison between D-loops; as expected considering the considerable sequence variability and difficulty in aligning these regions. Gene by gene diversity, as estimated by nucleotide divergence, was highly variable (Figure 4), and was highest in *nad4L* and *atp6*, and least in *rrnS*. The commonly used mitochondrial ‘barcodes’ for amphibians, partial *rrnL* (16S) [14,36] and partial *cox1* (COI) [37,38], the latter promoted by The Consortium for the Barcode of Life (http://www.barcodeoflife.org), are also indicated on Figure 4. Viewed in the context of overall nucleotide diversity, the 16S barcode captures less than COI, and both capture regions of relatively low sequence diversity across the mitochondrial genome. Novel molecular markers and barcodes that must achieve universality in their use across taxa, must have priming sites in conserved regions, as indicated by troughs in the graph; e.g. as seen at the 5’- and 3’-ends of the 16S barcode. Given that tRNA genes can move, duplicate or change identity (e.g. [39]), ideal priming sites should be within ribosomal or protein coding genes, and as gene order changes can occur, it may be preferable to target single gene fragments.

Given the unique interest in *Xenopus* as a model laboratory organism, it is necessary to consider hitherto unused regions of the mtDNA that may be of use as molecular markers. Populations of *Xenopus* used in labs worldwide are thought to have originated from different regions of Africa, and have been interbred, and indeed inbred, to varying degrees. Different populations could be identified through their maternal lineages via mitochondrial markers. Regions of high diversity may be of greatest utility here. The taxonomy of the group is also replete with subspecies and reliable markers are required for systematic revision as well as diagnosis.

Targeting regions of *Xenopus* mtDNA for novel molecular marker design depends very much on the intended application, and preferred technique. PCR-based amplifications of within-gene regions for bidirectional sequencing are a common starting point for differentiating individuals, populations and species. The sliding window analysis provides some regions worthy of pursuit. Although amongst the most variable of protein-coding genes, *nad4L* may be too short to be usable. However, alternative genes offering reasonable length (400-1000 bp) include *atp6*, *nad2* and *nad1.* By far the most variable regions of the mtDNAs is the D-loop. However, if this were to be a chosen target for within or between species study, it would likely need to be amplified in its entirety. High AT-content, the propensity for secondary structure folding and length differences make it difficult to design suitable PCR primers within the D-loop. However, fortuitously, two well-conserved genes (*cytb* and *rrnS*) border the D-loop and these offer many regions for potential PCR primer design.

Although none of the suggested markers were tested in this study, we found the software MitoMapper (Yang et al., 2011) was readily applicable to our xenopus data, yielding either suites of primer pairs to generate overlapping amplicons for complete *de novo* mtDNA coverage, or primer pairs for targeting shorter gene/genome regions (data not shown). The program designs primers that will work on the input sequences as well as other closely related taxa.

Combining the two new mitogenomes with those already available will also facilitate the design of novel molecular markers for resolving e.g. the phylogenies of pipids and amphibians as a whole and the design of conserved primers for long PCR and the assembly of next generation sequencing contigs.

#### dN/dS analysis

**Figure 5:**
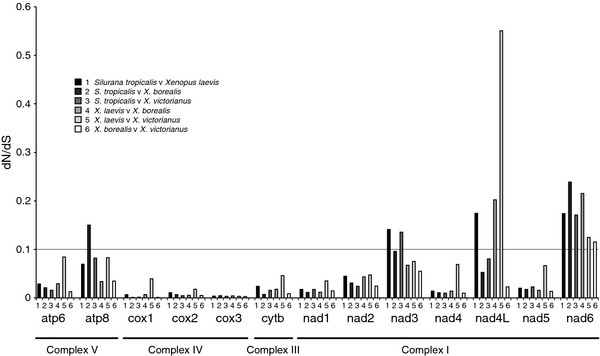
**Ratios of nonsynonymous/synonymous (dN/dS) nucleotide substitutions between the protein-coding genes of *xenopus *mitochondrial genomes.** Although the ratios differ considerably between genes, complexes and pairs of species, in all cases genes are evolving under negative (purifying) selective pressure (dN/dS < 1).

The ratio of nonsynonymous (dN) to synonymous (dS) substitutions observed within the *xenopus* protein-coding sequences suggests that all genes are evolving under negative (purifying) selection. Complex IV genes (*cox1-3*), the Complex III gene (*cyt* b), some of the complex I genes (*nd1, 2, 4* and *5*) and one of the complex v genes (*atp6*) are under strong selection, with the remaining complex I genes (*nad3*, *nad4L* and *nad6*) and one of the complex V genes (*atp8*) under weaker selection (Figure 5). These findings are concordant with study where dN/dS ratios were estimated from 347 complete vertebrate mitochondrial genomes, which included 54 from amphibians, that showed that purifying selection was strongest for genes that encode subunits with crucial functions in the RC [27]. Indeed, Complex IV subunits COX1-3 and Complex I subunits ND1, 2, 4 and 5 do have crucial functions in the RC. Specifically, the COX1 and COX2 subunits of Complex IV perform the electron and proton transfers, as well as creating the channels required for the dioxygen molecule to reach, and the H_2_O molecule to be removed from, the O_2_ reduction site [40]. While the COX3 subunits (Complex IV is a dimer) are also thought to be involved in proton transfers, they also provide structural stability between the COX1 and 2 subunits (reviewed in [41]). While the Complex I subunits ND5, ND4 and ND2 perform the proton pumping and ND1 provides structural stability between the membrane and peripheral domains of the complex. ND5 has an additional role as a “coupling element”, connecting all the membrane subunits together [42].

#### Expressed sequence tag analysis

**Figure 6:**
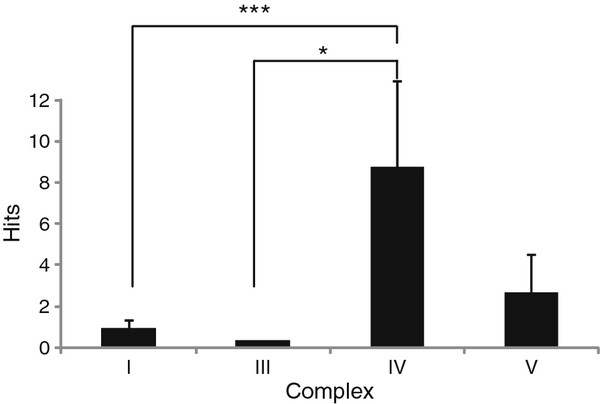
**Summary of expressed sequence tag database analyses of *S. tropicalis *protein coding sequences.** Mean (±s.e.m.) number of ESTs in Xenbase with ≥ 90% similarity to each of the 13 mitochondrial protein-coding sequences from ST. Individual gene sequences have been combined and are presented for each complex of the respiratory chain. ∗ = P <0.05 and ∗∗∗ = P <0.003 between complexes (as indicated).

In total, 78 ESTs with ≥90% similarity at the nucleotide level, to the protein coding genes of ST were recovered from the cDNA libraries deposited in Xenbase [43]. Complex IV (*cox1-3*) genes were significantly more represented than those of Complex I (*nad1-6* and *nad4L*) and the Complex III gene (*cytb*) (P < 0.003 and P < 0.05, respectively; Figure 6). Interestingly, Complex IV genes under the strongest purifying selection were also the most highly expressed. This correlation has been observed in several organisms, from bacteria to humans (reviewed in [27]). Given that Complex IV subunits perform such crucial functions in the RC and are expressed so highly, it is not really surprising that the genes that encode them contain few non-synonymous substitutions, relative to other genes. Preserving Complex IV gene function by purifying selection would avoid mutations that cause amino acid changes that could lead to the production of dysfunctional subunits and ultimately, a compromised RC. This is likely to be even more important if such genes are highly expressed. 

#### NGS approaches to mtDNA sequencing

In this study, two new *Xenopus* (*Xenopus borealis* and *X. victorianus*) complete mitochondrial genome sequences were obtained using a combination of long-PCR and next generation sequencing (454) of either individual or multiple pooled mtDNAs. This is the first report of amphibian mitochondrial genomes obtained using this approach. The relative ease with which the high quality genomes were obtained, due to: (1) the long-PCR step minimizing the amount of starting material (i.e. 10ng of egg total DNA) required and (2) the 454 sequencing step removing the need for an optimized primer set (necessary for a primer-walking strategy) and generating a high level of coverage (XB: 9823 and XV: 186; mean DOC), makes NGS a very promising approach for other amphibians. It is immediately applicable to other xenopus species/individuals, as all the primers used in this study were designed to be universal for xenopus mtDNAs. Furthermore, both approaches generate depths of coverage that far surpass traditional primer-walking strategies at either the same cost (~US$1400, approach 1) or less (~US$80, approach 2).

## Conclusions

Here we provide two approaches for the rapid and efficient amplification of amphibian mitogenomes from microgram quantities of tissue. Specifically, two new xenopus mitogenomes (*Xenopus borealis* and *X. victorianus*) were obtained, characterised and compared to their sister taxa (*X. laevis* and *Silurana tropicalis*), two of the most intensely studied amphibians and popular vertebrate model organisms. We discovered the size was similar and gene content and order was the same among the xenopus and to other vertebrates. The phylogeny, generated using amino acids, was consistent with existing phylogenies for xenopus and amphibian species, however, some 15 additional species of xenopus remain to analysed using this approach and would provide additional insights into their evolution and radiation. On the one hand we reveal commonly used mitochondrial “barcodes” for differentiating amphibian species and populations fail to capture the greatest xenopus nucleotide diversity, on the other hand we provide alternative, more appropriate targets for differentiating xenopus species/populations. Our exploration of protein-coding genes in the xenopus mitogenomes reveals their function is strongly preserved by negative (purifying) selection, particularly in the case of those encoding proteins that have crucial functions in the mitochondrial respiratory chain and are highly expressed, such as the Complex IV proteins: CO1-CO3, that collectively transfer protons and electrons or confer structural stability. Our complete mitochondrial genome amplification methods and analyses are applicable to other amphibians and are therefore likely to be helpful for identifying the most appropriate markers for differentiating species, populations and resolving phylogenies, a pressing need since amphibians are undergoing drastic global decline. Our findings also provide a platform for using xenopus to better understanding the critical role mitogenomes play in complex biological problems, such as cellular reprogramming, organogenesis, regeneration, gene regulatory networks and protein interactions that control growth and development.

## Methods

All reagents, materials and equipment were purchased from Sigma-Aldrich® (Gillingham, UK), unless stated otherwise.

### *Xenopus* egg collection and DNA extraction

One adult *Xenopus borealis* (XB) and *X. victorianus* (XV) female was injected with a priming dose (50 U) of human chorionic gonadotropin (hCG) and, one week later, an ovulatory dose (500 U) into the dorsal lymph sac. One day after injecting the ovulatory dose, a batch of eggs was collected manually into 1 X MBS (110 mM NaCl, 2 mM KCl, 1 mM MgSO4, 2 mM NaHCO3, 0.5 mM Na2HPO4, 15 mM Tris base, pH 7.6, acetic acid, 0.5 mM sodium phosphate, pH 7.4); dejellied using 2% (w/v) cysteine (in 1 X MBS) and washed three times with 1 X MBS. Groups of ten eggs (XB) or one egg (XV) were/was placed into 1.5 mL tubes, excess liquid removed and stored at -70°C. Total DNA was extracted from the eggs following thawing via: homogenisation in NETS buffer (0.3M NaCl, 1mM EDTA, 20 mM TRIS, pH 7.0), mixing the homogenate in phenol:chloroform:isoamyl alcohol 25:24:1 (PCIA) and centrifugation (13,000 rpm, 15 min). The aqueous layer (containing the DNA) was recovered and extracted with PCIA twice more prior to precipitating the DNA in ethanol at -20°C. DNA pellets were recovered from the ethanol via centrifugation (as above), air-dried and resuspended in 100 ul of nuclease-free water by heating for 1 h at 65°C.

### Long-PCR amplification of two mitochondrial genome regions

The complete mitochondrial genome of each *Xenopus* species was amplified by long-PCR as two amplicons (1: ~7,961bp, containing the *rrnL* and *cox1* genes and 2: ~9,649bp) using the Expand Long Range dNTPack kit (Roche). Each (50 μL) PCR contained 10 ng total DNA; 2 x buffer, 2.5 mM MgCl2, 0.5 μM each dNTP, 0.3 μM each of primers Long F1 and R2 (Amplicon 1) or Long F2 and R1 (Amplicon 2; Table 4 and Figure 2), 1.4% (v/v) DMSO and 0.7 μl of enzyme mix and was run on a GeneAmp® PCR system 9700 at 92°C for 2 min; 10 cycles at 92°C for 10 s, 55°C for 15 s, 68°C for 10 min; 20 cycles at 92°C for 10 s, 55°C for 15 s, 68°C for 10 min + 10 s per cycle; followed by 68°C for 7 min. Amplicons were resolved on 1% (w/v) agarose gels at 100V for 1 h, purified using the QIAquick® PCR purification Kit (QIAGEN, Hilden, Germany) and quantified using the Quant-iT™ PicoGreen® dsDNA assay Kit (Invitrogen) and a Spectramax microplate reader; Molecular Devices Ltd, Wokingham, UK).

**Table 4:** Primer details

**Name**	**Gene**	**Nucleotide position**	**Sequence (5’-3’)**	**Amplicon length (bp)**	**Annealing temp. (°C)**
LongF1	*trnF*_*atp6*/*atp6*	28-49	ACTGAAGATGCTGAGATGAGCC	7961	55
LongR2		8012-8033	ATGGTCAGTTTCAAGGGTTAGG		
LongF2	*atp6*/*atp6*_*trnF*	8012-8033	CCTAACCCTTGAAACTGACCAT	9649	55
LongR1		28-49	GGCTCATCTCAGCATCTTCAGT		
16SA-Lmod	*rrnL*	1943-1962	CGCCTGTTTACCAAAAACAT	580	53
16SA-H		2542-2562	CCGGTCTGAACTCAGATCACG		
COX1F	*cox1*	6613-6631	GAAACATGAGCAAAAATCC	190	53
COX1R		6821-6802	AATGCTTCTCAGATAATGAA		
ND6F	*nad6*	13996-14015	AACATCCCACCTAAATAAAT	106	53
ND6R		14137-14122	TAGCTGTTGCTTCAAATCC		
AMP1F	*trnF*_*rrnS*	5-23	ACGTAGCTTAAGTAAAGCACAGC	294	58
AMP1R		322-347	ATCAACTTGAGTTTCTCGTATAACC		
AMP2F	*cox2_trnK_atp6/cox3*	7776-7800	TCTTCATCAATACTAGAAGCCTCA	912	61
AMP2R		8712-8731	TGTGCTTGGTGTGCCATTA		

The names, gene and nucleotide positions, sequences, expected amplicon lengths and annealing temperatures of PCR primers used to generate the long-amplicons and to verify the specific identity of the long-amplicon and sequences of the primer regions.

### Verification of long-PCR amplicon identity and primer region sequences

Each (50 μL) PCR contained either 0.8 pg of amplicon or 20 ng total DNA; 1 x PCR Buffer (Bioline, London, UK), 1.5 mM MgCl_2_ (Bioline), 800 μM total dNTPs (Bioline), 0.5 μM each of primers 16S (F and R) or COXI (F and R; amplicon 1), Amp1 (F and R; primer region 1) or Amp2 (F and R; primer region 2), and 2.5 units BioTaq DNA polymerase (Bioline) and run on the same PCR machine as above at 95°C for 5 min; 35 cycles of 94°C for 30 sec, annealing temperature specific for each primer pair (Table 4) for 30 sec; 72°C for 30 sec, followed by 7 min at 72°C. PCR products were resolved on agarose gels, purified and quantified as before then sequenced the ABI BigDye® Terminator cycle sequencing kit v3.1 and the ABI PRISM 3730xl.

### Automated sequencing and assembly of the *Xenopus* mitochondrial genomes using 454

Amplicon 1 and 2 (15 μg of each) from XB were pooled and then used to construct a fragment library. Fragments were amplified by emulsion PCR, pyrosequenced on a PicoTiterPlateTM and detected via The Genome Sequencer FLX Titanium System^TM^ (service provided by Beckman Coulter Genomics, UK). Assembly of XB (and XV described below) used a two-step process, where a tentative consensus assembly was made first, and then original reads were mapped on to that consensus, thereby correcting it. The XB mitochondrial genome was assembled using Roche Newbler v 2.5.3 (Roche) and Mira v 3.2.1 [44]. Initially, 33 preliminary contigs from individual reads exceeding 520 bp were assembled. Contigs were exported as FASTA files and reassembled with Sequencher v. 4.10 (GeneCodes, Inc.), in order to visualise sequencing errors, indels and edit open reading frames, and to make a tentative consensus. Newbler was used to map the original reads back onto this tentative consensus, which made a few corrections to it. Although assembled initially as a linearized genome, Sanger sequences linking the two amplicons provided a fully resolved circular mitochondrial genome.

Amplicon 1 and 2 from XV were pooled (~0.5 pmol each) with an equimolar mixture of 470 long-PCR amplicons from a range of other species (>250 various metazoans most from different genera and only a few vertebrates; unpublished). A single D-phase library was constructed from the pooled samples and run on a FLX Titanium plate (service provided by Centre for Genomic Research, University of Liverpool). The mitochondrial genomes, among them XV, were assembled automatically using Mira v 3.2.1.17, with the "accurate" option [44]. In order to avoid chimeric assemblies from the multiplexed pool of amplicons, only reads > = 150 bp were chosen for the assembly. Putative XV contigs were identified by BLASTN, where the top hit was to a known *Xenopus* mt sequence. Those contigs were aligned to the *X. laevis* mitochondrial genome (HM991335) using BLASTn to make a tentative consensus of 17735 bp. All 9864 of the 454 reads that went into those contigs were extracted from as a separate sff file. Those reads were then mapped against the tentative consensus to make the final sequence. Both Mira and Newbler were trialled to do the mapping, but in this case Mira was used because Newbler introduced a frameshift in the *atp6* gene but Mira did not. The final mapping was 17731 bp long, using 6627 of the 9864 reads were used to make the consensus. The sequences were confirmed using Sanger tags, which had exact matches except for a few alignment gaps near the ends of the tags.

### Annotation and characterisation of tRNAs, rRNAs, D-loop and protein coding regions

Mitochondrial genomes of *Xenopus* were annotated using MacVector v. 12.0 (MacVector Inc.). Open reading frames were found employing the ‘vertebrate mitochondrial code’ and inferred translated proteins were confirmed by means of BLAST analysis. Initiation and termination codons were verified through comparison with published mtDNAs of *Xenopus* and other amphibians. The positions of all transfer RNA genes were identified using tRNAscan SE 1.21 [45] or ARWEN [46]. The rRNA genes and control region were identified by BLASTn analysis and comparisons with respective sequences within the XL and ST mitochondrial genomes.

### Phylogenetic, nucleotide variation and non-synonymous/synonymous substitution rate analysis of protein coding regions

Two early divergent members of the Pipidea: *Pipa carvalhoi* and *Hymenochirus boettgeri*, were selected as suitable outgroups. For protein coding genes only, the *Xenopus* and outgroup nucleotide sequences were aligned by eye, with reference to gene boundaries and were held in frame. All positions were unambiguously alignable. The alignment was translated into amino acids yielding 3,782 positions; none were excluded from the analysis as all could be aligned unambiguously. Phylogenetic trees were constructed using Bayesian inference (BI) with MrBayes, version 3.1.2 [47], employing the mixed amino acid model. Two runs, with four chains each (temp = 0.2), were run for 5,000,000 generations and sampled every 1000 generations; 500,000 generations were discarded as burn-in.

### Sliding window analysis

Sliding window analysis was performed on the aligned, complete mitochondrial genome nucleotide sequences of the four *Xenopus* species. Analyses were conducted on the full alignment, and from this also all combinations of pairwise comparisons between *Xenopus* species. Protein-coding genes were aligned in frame, as per the alignment conducted for the phylogenetic analysis. Intergenic regions, tRNA and rRNA genes were aligned by eye; although this was achieved with little ambiguity the region covering the D-loop could not be unambiguously aligned as a result of significant length and sequence differences. A sliding window of 300 bp and steps of 10 bp was used to estimate nucleotide divergence K(JC) between all species and between all pairs of species over the entire alignment using DnaSP v.5 [35]. Nucleotide divergence, for the entire and pairwise alignments, was plotted against midpoint positions of each window, and gene boundaries indicated.

Nonsynonymous (dN) and synonymous (dS) substitution between the *Xenopus* protein-coding sequences was estimated with KaKs Calculator using a modified version of the Yang-Nielsen algorithm, which is based on the Tamura-Nei Model that considers the difference among rates of transitional and transversional substitutions as well as factors in codon frequency bias [48]. dN and dS (or their ratio ω = dN/dS) are used to categories genes into three groups, those undergoing: negative (purifying) selection (ω < 1), positive (adaptive) selection (ω > 1), and neutral selection (ω = 1).

### Expressed sequence tag database mining of protein coding sequences

Each ST protein coding sequence was inputted into Xenbase [43], which contains ST expressed sequence tags (ESTs), from different developmental stages (unfertilised egg, cleavage, blastula, gastrula, neurula and tailbud), to look for clones containing mtDNA genes. Only matches with ≥ 90% similarity with the inputted sequence were reported.

## Competing interests

The authors declare that they have no competing interests.

## Authors’ contributions

RL conceived and designed the *Xenopus borealis* mitogenome sequencing, carried out the egg collections, DNA extractions, long-PCRs, short amplicon verifications and EST analyses. DTJL conceived and designed the *Xenopus victorianus* mitogenome sequencing, annotated both mitogenomes, and carried out phylogenetic, sliding window, and dN/dS analyses. RE and DTJL led on drafting the manuscript. PF assembled both *Xenopus* mitogenomes and helped draft the manuscript. MG oversaw the animal experiments and helped draft the manuscript. All authors read and approved the final manuscript.
